# Correction: Aslam et al. pH Sensitive Pluronic Acid/Agarose-Hydrogels as Controlled Drug Delivery Carriers: Design, Characterization and Toxicity Evaluation. *Pharmaceutics* 2022, *14*, 1218

**DOI:** 10.3390/pharmaceutics17010019

**Published:** 2024-12-26

**Authors:** Mariam Aslam, Kashif Barkat, Nadia Shamshad Malik, Mohammed S. Alqahtani, Irfan Anjum, Ikrima Khalid, Ume Ruqia Tulain, Nitasha Gohar, Hajra Zafar, Ana Cláudia Paiva-Santos, Faisal Raza

**Affiliations:** 1 Faculty of Pharmacy, The University of Lahore, Lahore 54000, Pakistan; maryyee994@hotmail.com (M.A.); anjuum95@yahoo.com (I.A.); 2 Faculty of Pharmacy, Capital University of Science and Technology (CUST), Islamabad 44000, Pakistan; nadiashamshad@gmail.com (N.S.M.); nitasha.gohar@cust.edu.pk (N.G.); 3 Department of Pharmaceutics, College of Pharmacy, King Saud University, Riyadh 11451, Saudi Arabia; msaalqahtani@ksu.edu.sa; 4 Faculty of Pharmaceutical Sciences, GC University, Faisalabad 38000, Pakistan; ikrima_khalid@yahoo.com; 5 Faculty of Pharmacy, University of Sargodha, Sargodha 40100, Pakistan; umeruqia_tulain@yahoo.com; 6 School of Pharmacy, Shanghai Jiao Tong University, 800 Dongchuan, Road, Shanghai 200240, China; hajrazafar@sjtu.edu.cn; 7 Department of Pharmaceutical Technology, Faculty of Pharmacy, University of Coimbra, 3000-548 Coimbra, Portugal; acsantos@ff.uc.pt; 8 REQUIMTE/LAQV, Group of Pharmaceutical Technology, Faculty of Pharmacy, University of Coimbra, 3000-548 Coimbra, Portugal

**Error in Figure**

In the original publication [1], there was a mistake in Figure 11 as published. Due to a technical error during the preparation of the manuscript, images from a different study conducted by the same research group were mistakenly included. The corrected Figure 11 appears below. The authors apologize for any inconvenience caused and state that the scientific conclusions are unaffected. This correction was approved by the Academic Editor. The original publication has also been updated.

## Figures and Tables

**Figure 11:**
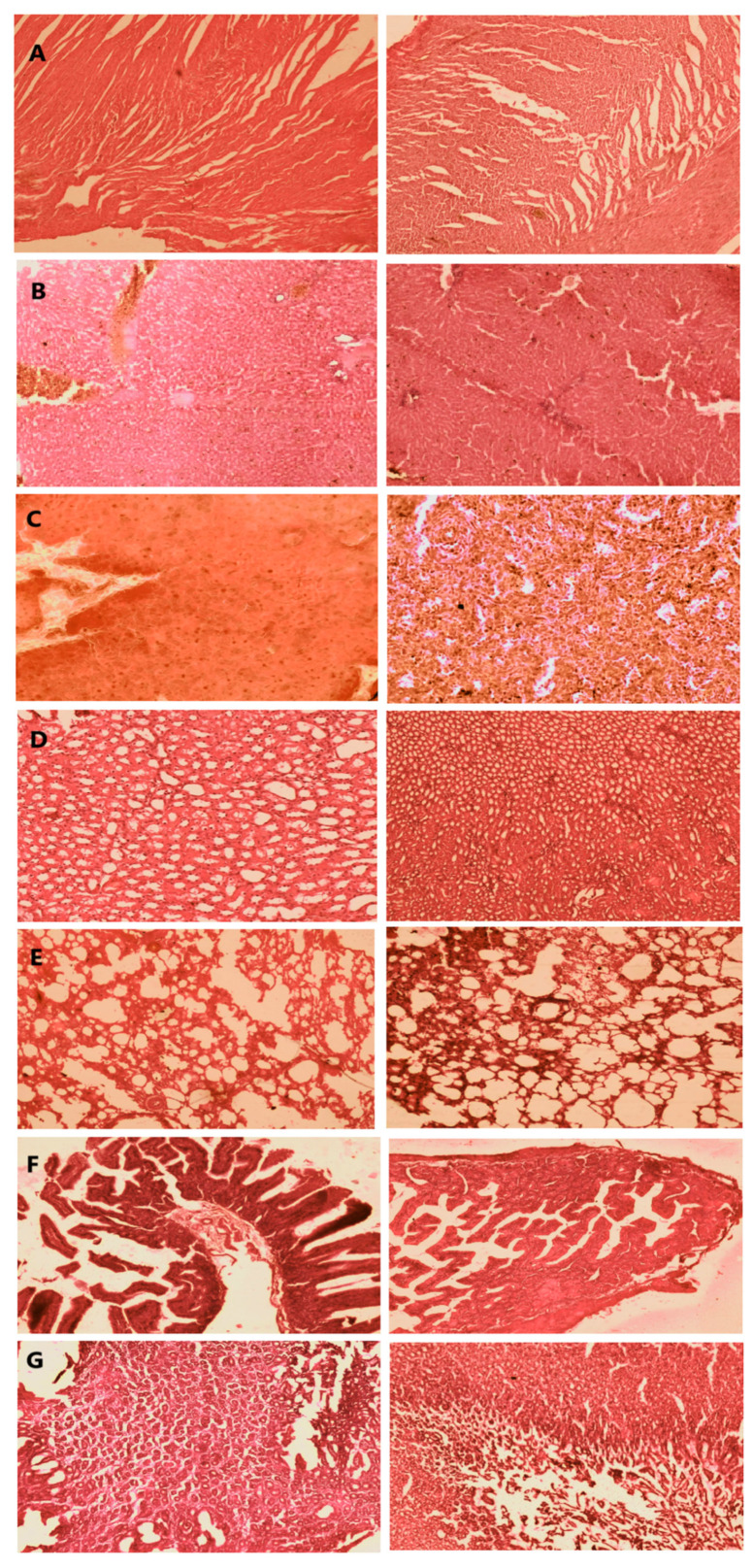
Micrographs of control and test group of rabbit’s organ tissues ((**A**) heart tissue, (**B**) liver, (**C**) spleen, (**D**) kidney, (**E**) lungs, (**F**) intestine, and (**G**) stomach).
